# Vitamin D and Mean Platelet Volume as Biomarkers in Pediatric Obstructive Sleep Apnea: Associations with Disease Severity and Sleep Parameters

**DOI:** 10.3390/jcm15051878

**Published:** 2026-03-01

**Authors:** Anna Di Sessa, Giuditta Bargiacchi, Ludovica Nucci, Giovanni Messina, Letizia Perillo, Maria Esposito, Marco Carotenuto, Maria Ruberto

**Affiliations:** 1 Department of Woman, Child, and General and Specialized Surgery, University of Campania “Luigi Vanvitelli”, 80138 Naples, Italy; 2 Clinic of Child and Adolescent Neuropsychiatry, Department of Mental Health, Physical and Preventive Medicine, University of Campania “Luigi Vanvitelli”, 80131 Naples, Italy; giuditta.bargiacchi@studenti.unicampania.it (G.B.); ludovica.nucci@unicampania.it (L.N.); maria.esposito3@unicampania.it (M.E.); marco.carotenuto@unicampania.it (M.C.); 3 Department of Experimental Medicine-Section of Human Physiology and Unit of Dietetics and Sports Medicine, University of Campania “Luigi Vanvitelli”, 80138 Naples, Italy; giovanni.messina@unicampania.it; 4 Multidisciplinary Department of Medical-Surgical and Dental Specialties, University of Campania “Luigi Vanvitelli”, 80138 Naples, Italy; letizia.perillo@unicampania.it; 5 Department of Education and Sport Sciences, Pegaso Telematic University, 80143 Naples, Italy; maria.ruberto@unipegaso.it

**Keywords:** pediatric obstructive sleep apnea, vitamin D, mean platelet volume, biomarker, sleep architecture

## Abstract

**Background/Objectives**: Vitamin D and mean platelet volume (MPV) have been suggested as biomarkers of obstructive sleep apnea (OSA) in adults, while pediatric data remain limited. We aimed (i) to investigate associations between vitamin D and MPV with the presence and severity of pediatric OSA and (ii) to explore potential associations between vitamin D status and sleep parameters in normal-weight affected children. **Methods**: A total of 138 children with polysomnography-confirmed OSA and 138 age- and sex-matched controls were enrolled. All participants underwent detailed clinical, biochemical, and overnight sleep assessments. The OSA group was stratified according to disease severity. **Results**: Vitamin D levels were significantly lower in OSA patients (*p* < 0.0001), while MPV, C-reactive protein (CRP), and erythrocyte sedimentation rate (ESR) were significantly higher (all *p* < 0.0001) than in the controls. Severe OSA was associated with elevated inflammation markers, higher insulin resistance, and lower vitamin D levels (all *p* < 0.0001). Vitamin D levels were inversely associated with the Apnea–Hypopnea Index (AHI) (R 0.37, adjusted r^2^ 0.13, *p* < 0.0001) and Oxygen Desaturation Index (ODI) (R 0.36, adjusted r^2^ 0.13, *p* < 0.0001), even after adjustments (both *p* < 0.0001). N1 and REM sleep were negatively associated with vitamin D in the OSA group (*p* = 0.01 and *p* = 0.02, respectively). Vitamin D deficiency was independently associated with higher odds of OSA (adjusted OR = 6.76, 95% CI: 3.97–11.51, *p* < 0.0001). Similarly, OSA presence was associated with lower vitamin D levels (aOR = 1.40, 95% CI: 1.06–1.94, *p* = 0.03). **Conclusions**: Vitamin D and MPV are associated with the presence and severity of pediatric OSA. Vitamin D levels were related to specific sleep architecture parameters, and MPV appeared to reflect inflammation associated with OSA, supporting their potential utility as biomarkers in pediatric OSA.

## 1. Introduction

Pediatric obstructive sleep apnea (OSA) is a common sleep disorder characterized by prolonged partial upper airway obstruction and/or intermittent complete obstruction (obstructive apnea). These events disrupt normal nocturnal ventilation and sleep architecture [1,2,3]. The prevalence of OSA in children is increasing globally, with epidemiological data indicating that it peaks between the ages of 2 and 8 years [4]. Its general prevalence varies depending on the diagnostic criteria used, ranging from 0.1% to 13% when including parent-reported surveys, and from 1% to 4% when relying on objective indicators such as polysomnography (PSG) or pulse oximetry [1,5].

Notably, OSA is associated with both an unfavorable cardiometabolic risk profile [1,6,7,8] and cognitive impairments [1,9,10]. This dual impact highlights the complex etiology of the disorder [7,11,12].

Several risk factors for OSA have been identified, including obesity, craniofacial abnormalities, impaired upper airway muscle function, nocturnal fluid shifts, pharyngeal neuropathy, and bronchial asthma [1,4,12,13]. However, the precise pathophysiological mechanisms underlying the condition remain unclear [1]. Alterations in various signaling pathways are thought to contribute to OSA, leading to oxidative stress, systemic inflammation, insulin resistance (IR), and sympathetic activation, which may result in a range of systemic diseases such as cardiovascular, metabolic, and neurological disorders [1,4].

Vitamin D has emerged as a central player in the complex pathophysiology of OSA [12,14,15], although evidence remains conflicting [15,16,17].

Owing to its pleiotropic actions, vitamin D is increasingly implicated in OSA pathophysiology [14,18,19]. Beyond its classical role in calcium and bone homeostasis [19], vitamin D modulates immune function and reduces pro-inflammatory cytokine expression through pathways such as NF-κB and MAPK signaling [19,20]. Moreover, vitamin D influences oxidative stress and mitochondrial function. Deficiency is associated with increased reactive oxygen species and impaired oxidative homeostasis in skeletal muscle [20]. Vitamin D also plays a role in neuromuscular function and muscle metabolism, and deficiency has been linked to reduced muscle strength and altered intracellular calcium handling, which may adversely affect muscle contractility [20]. Consequently, vitamin D deficiency may promote chronic upper airway inflammation and lymphoid tissue proliferation. This could lead to adenotonsillar hypertrophy and increased upper airway collapsibility [21]. Low vitamin D status has also been associated with metabolic dysregulation and IR [20,22], recognized contributors to cardiometabolic dysfunction in OSA [1]. Taken together, these mechanisms provide a biological rationale for an association between vitamin D deficiency and OSA [14,18,19], although causality remains to be fully elucidated, especially in children [14,15]. Notably, recent studies in adults have suggested a negative role of vitamin D deficiency in sleep architecture [14,20,21,23], but similar pediatric data remain limited [15].

Considering the cardiometabolic burden of OSA and its long-term impact on health outcomes in individuals with obesity as well as in normal weight [1,8], early diagnosis is essential [2]. While PSG remains the diagnostic gold standard for OSA [2], its availability is often limited in routine practice. This has motivated research into alternative diagnostic tools and accessible biomarkers [24], including vitamin D levels and mean platelet volume, for OSA detection and staging [21,25,26]. Given its association with systemic inflammation—a key pathophysiological feature of OSA [1,5]—mean platelet volume (MPV) has been proposed as a biomarker for OSA diagnosis and disease staging in adults [27,28,29,30]. OSA is characterized by recurrent episodes of intermittent hypoxia and chronic inflammation [1], which promote increased platelet production and activation, ultimately leading to elevated MPV levels [30]. Numerous adult studies have reported associations between MPV values, OSA severity, and related cardiovascular risk [29,30]. In contrast, although emerging data in pediatric patients appear promising, evidence in children remains limited [30,31].

To fill this gap, we aimed (i) to investigate associations between vitamin D and MPV with the presence and severity of pediatric OSA and (ii) to explore potential associations between vitamin D status and sleep parameters in normal-weight affected children.

## 2. Materials and Methods

### 2.1. Study Population

A total of 138 children diagnosed with OSA through a full nocturnal PSG and consecutively referred to the Pediatric Sleep Lab of Clinic of Child and Adolescent Neuropsychiatry at the University of Campania “Luigi Vanvitelli” from October 2014 to November 2020 were enrolled. A control group of 138 non-OSA children recruited from the general pediatric outpatient clinics of the Campania Region was also included.

Exclusion criteria were considered as follows: (i) endocrine diseases (e.g., diabetes mellitus), (ii) neurological disorders (i.e., epilepsy, neuromuscular disorders, cerebral palsy), (iii) psychiatric symptoms (including attention deficit hyperactivity disorder, depression, and behavioral problems), (iv) mental retardation (IQ ≤ 70), (v) borderline intellectual functioning (IQ ranging from 71–84), (vi) overweight (body mass index (BMI) ≥85th percentile) or obesity (BMI ≥ 95°percentile), (vii) allergies, (viii) consumption of medications known to affect vitamin D metabolism, platelet indices, or sleep architecture, including vitamin D supplementation, corticosteroids, antiepileptic drugs, antifungal agents, anticoagulants or antiplatelet drugs, chronic sedative or hypnotic medications, and psychoactive drugs, (ix) systemic conditions known to affect vitamin D metabolism or absorption (e.g., chronic kidney and liver diseases, inflammatory bowel diseases, including Crohn’s disease, celiac disease, malabsorption syndromes, and other gastrointestinal disorders, etc.), (x) missing data or (xi) denying consent to undergo diagnostic procedures.

The Ethical Institutional Review Board of our University approved the study (protocol code 776/2014, date of approval 31 July 2014). The study was conducted according to the ethical guidelines of the 1975 Declaration of Helsinki and its later amendments or comparable ethical standards. Written informed consent was obtained from parents before any procedure.

All the enrolled subjects underwent a comprehensive clinical and laboratory evaluation. Anthropometric indices, including body mass index standardized for age and sex (BMI-SDS), were measured according to standard methods, as previously reported [8].

To assess the adherence of the whole diet of the study participants of both cohorts to the Mediterranean diet model and its potential link with serum vitamin D levels, the Mediterranean Diet Quality Index for children and adolescents (KIDMED) was used by measuring the consumption of 16 components [32]. Questions denoting a negative connotation with respect to the Mediterranean diet (i.e., fast foods, baked goods, sweets, etc.) were assigned a value of −1, and those with a positive aspect (i.e., fruit, vegetables, cereals, nuts, etc.) were scored +1. According to the KIDMED index, a score of 0–3 reflects a poor adherence to the Mediterranean diet, a score of 4–7 describes average adherence, and a score of 8–12 a good adherence [32].

### 2.2. Laboratory Evaluation

After an overnight fast, blood samples were drawn to measure the main biochemical and metabolic parameters [8]. Fasting glucose (mg/dL), fasting insulin (µU/mL), uric acid (mg/dL), and liver enzyme levels (U/L) were measured using standardized laboratory procedures.

Insulin resistance was estimated using the homeostasis model assessment for insulin resistance (HOMA-IR), calculated as fasting insulin (µU/mL) × fasting glucose (mg/dL)/405 [8]. Serum vitamin D concentrations (ng/mL) were also measured [22].

Inflammatory markers included mean platelet volume (MPV, fL), C-reactive protein (CRP, mg/dL), and erythrocyte sedimentation rate (ESR, mm/h) [8]. MPV was measured in blood collected into K2-EDTA tubes and analyzed within 1 h of collection to minimize pre-analytical variability related to anticoagulant type, storage, or time to analysis. All samples were processed under consistent procedures to ensure reliability and reduce potential measurement bias.

### 2.3. Sleep Evaluation

All study participants, including both the OSA and control groups, underwent full overnight PSG using a standardized protocol [8,33].

The electroencephalogram (EEG) recordings and electrode placement were performed according to the 10–20 electrode system of the international federation Electroencephalography and Clinical Neurophysiology and the PSG montage included at 19 EEG channels (e.g., Fp2, Fp1, F3, F4, F7, F8, C3, C4, T3, T4, P3, P4, T5, T6, O1, O2, Fz, Cz, Pz), referenced to the contralateral mastoid, left and right electrooculogram (EOG), chin electromyogram (EMG), left and right tibialis EMG, electrocardiogram (ECG) (1 derivation), nasal cannula, thorax and abdominal effort, peripheral oxygen saturation, pulse and position sensors.

PSGs were carried out using a Brain Quick Micromed System Plus recording machine (Micromed S.p.A., Mogliano Veneto, Italy), and signals were sampled at 256 Hz and stored on a hard disk for further analysis [8]. EEG signals were digitally band-pass filtered at 0.1–120 Hz with 12-bit A/D precision. Sleep signals were sampled at 200 or 256 Hz and stored on a hard disk in European data format (EDF), a simple format for the exchange of digitized polysomnographic recordings for further analysis. EEG signals, in particular, were first acquired with a wide-band analogue filter (0.001–70 Hz) and then digitally band-pass filtered at 0.1–50 Hz. All recordings started at the habitual bedtime of the subjects and continued until a spontaneous morning awakening.

Sleep was subdivided into 30 s epochs, and sleep stages were scored according to the standard criteria [34]. The following conventional sleep parameters were evaluated: time in bed (TIB); sleep period time (SPT): time from sleep onset to sleep end; total sleep time (TST): time from sleep onset to the end of the final sleep epoch minus time awake; sleep latency (SL): time from lights out to sleep onset, defined as the first of two consecutive epochs of sleep stage 1 or one epoch of any other stage, in minutes; REM latency (RL): time from sleep onset to the first REM sleep epoch; number of stage shifts/hour (SS/h); number of awakenings/hour (AWN/h); sleep efficiency (SE%): the percentage ratio between total sleep time and time in bed (TST/TIB × 100); percentage of SPT spent in wakefulness after sleep onset (WASO%), i.e., the time spent awake between sleep onset and end of sleep; and percentage of SPT spent in sleep stages 1 (N1%), 2 (N2%), slow-wave sleep (N3%), and REM sleep (REM%). Additionally, the Oxygen Desaturation Index (ODI) was calculated as the average number of ≥3% oxygen desaturations per hour of sleep, reflecting the frequency of intermittent hypoxemia during the night [34].

All PSG recordings were visually scored by a trained investigator using Hypnolab 1.2 sleep software (SWS Soft, Troina, Italy), with sleep parameters tabulated for statistical analysis.

Respiratory events, sleep stages, and arousals were scored according to the American Academy of Sleep Medicine (AASM) Manual for the Scoring of Sleep and Associated Events, pediatric criteria, Version 3, 2023 [34].

Obstructive apnea was defined as a ≥90% reduction in airflow for at least two breaths with continued respiratory effort, while central apnea was defined as cessation of airflow for ≥2 breaths without respiratory effort [34]. Hypopnea was defined as a ≥50% reduction in airflow for ≥2 breaths associated with either ≥3% oxygen desaturation or an arousal [34]. Respiratory effort-related arousals (RERAs) were identified based on flow limitation with EEG arousal without meeting criteria for apnea or hypopnea [34]. The Apnea–Hypopnea Index (AHI) was calculated as the average number of apneas, hypopneas, and RERAs per hour of sleep [34]. OSA severity was classified as mild (AHI 1–<5 events/h), moderate (AHI 5–<10 events/h), or severe (AHI ≥ 10 events/h) according to international pediatric guidelines [34].

The control group included children with an Apnea–Hypopnea Index (AHI) < 1 event/hour, confirming the absence of sleep-disordered breathing. The OSA group included children across all severity levels (mild, moderate, and severe) according to standard pediatric PSG criteria [33,34].

### 2.4. Vitamin D Measurement

After an overnight fast, serum 25-hydroxyvitamin D [25(OH)D] concentrations were determined from a single blood sample collected between 08:00 and 09:00 a.m. [8,22]. Quantification was performed using a chemiluminescent immunoassay (CLIA; Liaison, DiaSorin, Saluggia, Italy) following standardized laboratory protocols. Vitamin D status was categorized [31] as sufficient (>30 ng/mL; >75 nmol/L), insufficient (20–30 ng/mL; 50–75 nmol/L), or deficient (<20 ng/mL; <50 nmol/L) based on current pediatric reference ranges [35]. To minimize potential confounding due to seasonal and environmental factors, sample collection was distributed across different seasons, and all samples were obtained in the morning to account for diurnal fluctuations in serum 25-(OH)D levels.

### 2.5. Statistical Analysis

Continuous variables were assessed for normality using the Shapiro–Wilk test. For two-group comparisons (e.g., OSA vs. controls), normally distributed variables were analyzed using the independent-sample *t*-test, whereas non-normally distributed variables were analyzed with the Mann–Whitney U test. For comparisons involving more than two groups (e.g., OSA severity: mild, moderate, and severe), normally distributed variables were analyzed using one-way ANOVA, and non-normally distributed variables were analyzed using the Kruskal–Wallis test. When significant differences were detected, post hoc pairwise comparisons were performed using Tukey’s HSD (for ANOVA) or Mann–Whitney U tests with Bonferroni correction (for Kruskal–Wallis) to control for Type I error.

Non-normally distributed variables were log-transformed prior to parametric analyses, including general linear models (GLMs), linear models and logistic regression models, to meet normality assumptions. Non-parametric analyses (Mann–Whitney U test) were performed on non-normal variables.

Categorical variables were compared using the chi-squared test.

The main features were compared between the OSA group and the controls. Within the OSA group, patients were further categorized according to disorder severity.

Relationships between sleep parameters and vitamin D levels were evaluated using linear regression analyses, adjusting, when appropriate, for key covariates including age, sex, BMI-SDS, and HOMA-IR, which were selected based on known associations with both OSA and vitamin D metabolism [15]. Dietary quality, assessed using the KIDMED score, was also considered as a potential covariate. However, as it showed no significant association with vitamin D levels, it was not included in the regression models.

Logistic regression analyses were conducted to assess the association between OSA and the odds of vitamin D deficiency, with results expressed as odds ratios (ORs) and 95% confidence intervals (CIs).

A GLM was used to examine the variance in vitamin D levels in the study population, with the same covariates included. Non-normally distributed variables were log-transformed prior to inclusion in parametric models.

IBM SPSS Statistics software, Version 24 (IBM, Armonk, NY, USA), was used for all statistical analyses. For interpretability, the results are presented as raw means ± standard deviations (SDs).

A two-tailed *p*-value less than 0.05 was considered statistically significant.

## 3. Results

**Figure 1:**
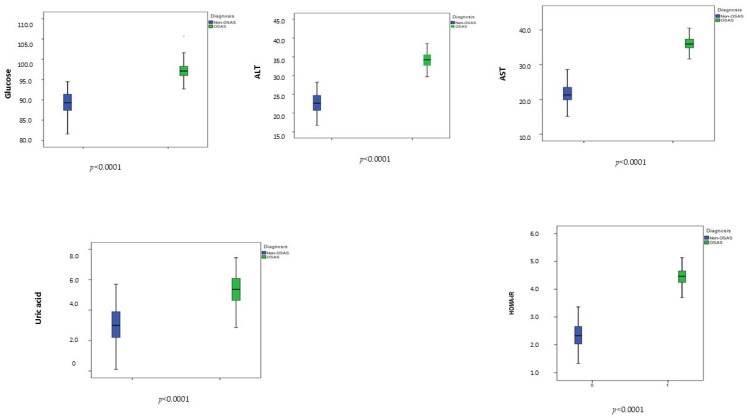
Distribution of the main cardiometabolic parameters between patients and controls. Abbreviations: ALT, alanine transaminase; AST, aspartate transaminase; HOMA-IR, homeostasis model assessment of insulin resistance.

The study population showed a mean age of 9.03 ± 1.32 years and a mean BMI-SDS of 0.65 ± 0.20. Patients with OSA (*n* = 138) exhibited higher BMI-SDS values than controls (0.67 ± 0.14 vs. 0.62 ± 0.25, *p* = 0.049). As expected, children with OSA showed an overall worse cardiometabolic profile, including higher fasting glucose (mg/dL), HOMA-IR, uric acid (mg/dL), and liver enzyme levels (U/L) compared with controls (all *p* < 0.0001) (Figure 1).

**Figure 2:**
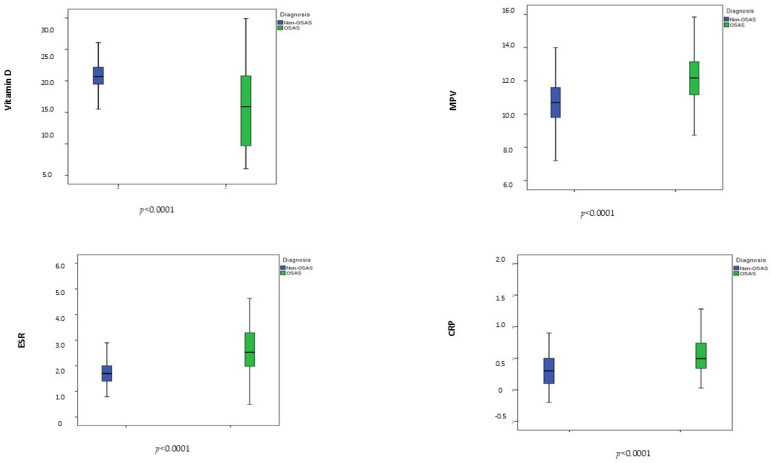
Distribution of vitamin D and of inflammatory marker parameters between patients and controls. Abbreviations: CRP, C-reactive protein; ESR, erythrocyte sedimentation rate; MPV, mean platelet volume.

Serum vitamin D concentrations (ng/mL) were significantly lower in patients with OSA than in controls (15.90 ± 6.06 vs. 20.87 ± 2.23, *p* < 0.0001). Regarding inflammatory markers, MPV (fL), C-reactive protein (CRP, mg/dL), and erythrocyte sedimentation rate (ESR, mm/h) were significantly higher in the OSA group compared with controls (12.25 ± 1.46 vs. 10.67 ± 1.24, *p* < 0.0001; 0.56 ± 0.33 vs. 0.31 ± 0.24, *p* < 0.0001; 2.60 ± 0.94 vs. 1.70 ± 0.42, *p* < 0.0001, respectively). (Figure 2).

No significant differences in nutritional status were observed based on KIDMED scores (all *p* > 0.05).

As expected, polysomnographic evaluation revealed increased WASO (minutes), AWN-h (events/hour), and N1 values (%), along with reduced REM sleep percentage, in children with OSA compared with controls (all *p* < 0.0001).

The principal features of the OSA group, subdivided according to disease severity, are presented in Table 1. MPV, CRP, and ESR levels were significantly higher in patients with a severe form of OSA than in those presenting with milder forms (all *p* < 0.0001). As expected, decreased serum vitamin D and higher HOMA-IR values were also found in patients with severe OSA compared to others (*p* < 0.0001).

A significant inverse association between AHI and vitamin D levels was found in the study population (R 0.37, adjusted r^2^ 0.13, *p* < 0.0001). Similarly, ODI values were inversely associated with vitamin D levels (R −0.36, adjusted r^2^ 0.13, *p* < 0.0001). Both associations remained statistically significant even after adjustment for confounding factors (both *p* < 0.0001).

A GLM for vitamin D variance (including sex, age, BMI-SDS, and HOMA-IR) in the study population confirmed the inverse association with OSA (r^2^ 0.19, adjusted r^2^ 0.17, *p* model < 0.0001) (Table 2). The same analysis conducted in the OSA group demonstrated a negative association with OSA severity (r^2^ 0.19, adjusted r^2^ 0.17, *p* < 0.001) (Table 3).

N-1 min was negatively correlated with vitamin D levels (R −0.29, adjusted r^2^ 0.05, *p* = 0.01) in the OSA group. This association was also confirmed in a GLM analysis adjusted for confounders (adjusted r^2^ 0.05, *p* = 0.02). The same analysis performed in the control group was not significant (adjusted r^2^ 0.006, *p* = 0.32).

REM-min was negatively correlated with vitamin D levels (R −0.28, adjusted r^2^ 0.05, *p* = 0.02) in the OSA group, while no significant correlation was found in the control group (adjusted r^2^ 0.01, *p* = 0.23).

Patients with OSA exhibited an adjusted odds ratio (aOR) for age, sex, BMI-SDS, and HOMA-IR to show vitamin D deficiency of 1.40 (95% CI:1.06–1.94; *p* = 0.03). Patients with vitamin D deficiency showed an aOR to show OSA of 6.76 (95% CI:3.97–11.51; *p* < 0.0001).

## 4. Discussion

Our findings indicate that children with OSA exhibit lower vitamin D levels and higher MPV values, particularly in more severe forms, highlighting the potential utility of these markers for diagnosing and staging the disorder. We also observed that children with OSA have a higher risk of vitamin D deficiency and experience greater sleep fragmentation compared to their non-OSA peers, reinforcing and extending adult evidence of the association between OSA, vitamin D status, and sleep architecture [23].

In line with previous evidence [8], our findings also confirmed an unfavorable cardiometabolic profile in affected normal-weight children. In this context, children with OSA exhibited significantly higher ESR and CRP levels compared with controls. However, despite reaching statistical significance, the absolute values of these markers remained within normal ranges and below thresholds for overt systemic inflammation. This pattern is indicative of subclinical, low-grade inflammation rather than frank inflammatory disease, consistent with previous pediatric studies reporting modest elevations of inflammatory biomarkers in this population. Nevertheless, even this low-grade inflammatory activity may contribute to the early cardiometabolic and vascular alterations observed in children with OSA [36,37].

Although all children in our study were within the normal-weight range, OSA patients had slightly higher BMI-SDS than controls (0.67 vs. 0.62, *p* = 0.049), indicating a marginal but statistically significant difference. While the absolute difference is small, even subtle variations in adiposity may influence cardiometabolic parameters such as blood pressure, IR, uric acid, and liver enzymes, as previously reported in normal-weight children. To address this potential confounding, we adjusted our analyses for BMI-SDS and other key covariates. The observed associations remained statistically significant after adjustment, indicating that they are independent of any residual differences in adiposity.

Recent studies have established an association between vitamin D deficiency and OSA in adults, with emerging evidence in pediatric populations [17,38].

Vitamin D, a fat-soluble secosteroid, plays a crucial role in bone development and the maintenance of mineral homeostasis [19,20]. Beyond its well-established function in skeletal health [20], vitamin D is increasingly recognized for its involvement in numerous physiological processes, owing to the presence of its receptors in the majority of body cells and tissues, underscoring its extensive role in maintaining overall health [20]. Indeed, it modulates the immune response, regulates gene expression, and influences cellular proliferation and differentiation [17,39]. Over recent decades, a growing body of evidence has associated vitamin D deficiency with a wide range of adverse health outcomes, including impaired immune regulation, allergic conditions, cardiovascular disease (CVD), various cancers, infectious diseases, dysregulated glucose metabolism, IR, obesity, diabetes, and sleep disturbances [15,40,41]. In particular, vitamin D has been implicated in the regulation and maintenance of sleep, suggesting a potential mechanistic role in sleep disorders [14,15,39,41].

Interestingly, a notable link between OSA and vitamin D deficiency has been demonstrated, with severity-dependent reductions in vitamin D levels [15,41,42]. In adults, a complex interrelationship among vitamin D, OSA, and cardiometabolic health has been reported [40], highlighting the potential prognostic significance of vitamin D in this context [40,41].

Both OSA and its severity have been widely linked to vitamin D deficiency [15,16,18], but the causal relationship and potential therapeutic implications remain unclear [15,39]. Although several studies suggest that vitamin D can benefit OSA management [14,16,17], the underlying mechanisms of this association are not fully understood [39,41]. Putative theories include vitamin D involvement in inflammation, hypoxia, immune response, muscle function, and vitamin D receptor gene polymorphisms [38,40]. The direction of the relationship—whether vitamin D deficiency contributes to OSA or vice versa—remains debated [39,41,42]. Limited evidence suggests an independent association between vitamin D and intermittent hypoxia [15], while other studies have linked persistent vitamin D deficiency to upper airway muscle dysfunction and adenotonsillar hypertrophy, which may contribute to OSA development in adults [41,42]. Although research on vitamin D and sleep disorders in children is sparse [15,38], there is emerging evidence on the impact of vitamin D levels on sleep architecture in this age group [15].

However, most available data supporting a positive correlation between vitamin D levels and sleep architecture, as measured by actigraphy, PSG, and questionnaires, are from cross-sectional studies in adults [23].

Of note, OSA is associated with an unfavorable cardiometabolic risk profile in both adults and children [8,40,41], and vitamin D deficiency is similarly recognized as a risk factor for cardiovascular morbidity and mortality [40,41]. Both conditions share common risk factors, such as age, obesity, and cardiometabolic comorbidities [2,40]. In this context, vitamin D status may serve as a mediator or explain the association between OSA and cardiometabolic morbidity [40,41], though further research is needed in this area [41]. Interestingly, supplementation of vitamin D has shown potential to improve sleep architecture [38,39], further supporting the link between insufficient vitamin D levels and sleep disorders [15,38,39], yet causal pathways remain to be established [1,39].

In our study, vitamin D levels were inversely associated with AHI (R = −0.37, adjusted r^2^ = 0.13, *p* < 0.0001) and ODI (R = −0.36, adjusted r^2^ = 0.13, *p* < 0.0001). We also observed statistically significant but modest negative correlations between vitamin D levels and N1 and REM percentages in the OSA group (N1-min: R = −0.29, adjusted r^2^ = 0.05, *p* = 0.01; REM-min: R = −0.28, adjusted r^2^ = 0.05, *p* = 0.02), while no significant associations were detected in controls. Although these associations remained statistically significant after adjustment for key confounding factors, the relatively low adjusted r^2^ values indicate that only a limited proportion of the variance in sleep parameters is explained by vitamin D levels, highlighting the multifactorial determinants of vitamin D status in pediatric OSA [15,17,41]. These findings, particularly for N1 and REM, should be considered exploratory, due to the small effect sizes and potential residual confounding, and should not be overinterpreted at the individual level. Pediatric sleep architecture is influenced by a complex interplay of developmental and environmental factors, which may attenuate or obscure the strength of associations with vitamin D. Therefore, while these findings are meaningful at the population level, their predictive value for individual patients is limited.

Considering the link between OSA and cardiometabolic health [8,40], there is a pressing need for easily accessible biomarkers to facilitate early diagnosis and staging of the disorder. MPV, a marker of platelet activation, has emerged as a promising marker of OSA diagnosis and severity in adults [27,29], likely due to the chronic intermittent hypoxia, oxidative stress, and systemic inflammation characteristic of OSA, which enhance platelet activation and aggregation [28]. Increased platelet reactivity contributes to a prothrombotic state and may underlie the elevated CVD risk observed in previous studies [7,28,29]. Several studies have reported elevated MPV in adults with OSA, supporting its potential as a simple and accessible biomarker for identifying patients at higher CVD risk [28,29].

However, in pediatric populations, evidence on MPV in OSA is still limited and somewhat inconsistent [30,31,43]. Some studies have reported higher MPV values in children with OSA compared with controls [30,31], with reductions observed after adenotonsillectomy [44], suggesting a potential link with platelet activation and cardiovascular risk [30,31]. Moreover, a recent study examining 190 children with OSA found positive correlations between MPV and AHI and negative correlations with lowest oxygen saturation [45], further supporting a link between MPV and disease severity in pediatric OSA [30]. By contrast, some evidence demonstrated no significant association between MPV and OSA, and in certain cases, even lower MPV in more severe disease [43].

Nevertheless, pediatric studies are limited. Further well-controlled research is needed to clarify the clinical utility of MPV in this population [30]. It is important to acknowledge that, despite standardized pre-analytical handling, MPV exhibits inter-laboratory variability, which may constrain the generalizability of these findings beyond our center.

The strengths of this study include the large, well-phenotyped cohort of normal-weight children with OSA, including a comprehensive polysomnographic evaluation that measures both respiratory and sleep parameters. Additionally, the availability of a control group with thorough clinical, laboratory, and polysomnographic evaluations enhances the robustness of this study. Nevertheless, although PSG-confirmed healthy controls provide a rigorous comparison, they may not fully reflect the general pediatric population, potentially limiting external validity. Several other limitations should also be acknowledged. The single-center, cross-sectional design precludes causal inference and may limit generalizability. Furthermore, certain variables that may influence both vitamin D status and OSA severity—including detailed dietary intake, physical activity levels, sunlight exposure, and socioeconomic status—were not directly assessed and may have contributed to residual confounding despite adjustment for key covariates (age, sex, BMI-SDS, and HOMA-IR).

Additionally, although vitamin D levels are influenced by seasonal variation, sunlight exposure, and outdoor activity, blood samples were collected throughout the year in the morning to minimize both diurnal and seasonal variability. Nevertheless, direct assessment of individual sunlight exposure and outdoor activity was unavailable, and residual confounding—including potential effects of seasonality on between-group differences—cannot be entirely excluded.

Finally, although appropriate statistical methods and post hoc corrections were applied, the relatively large number of comparisons performed may increase the risk of Type I error. Accordingly, some observed associations, particularly those with modest effect sizes, should be interpreted with caution. Replication in independent cohorts, as well as confirmation through longitudinal and interventional studies, is needed to strengthen robustness and clinical relevance.

Future investigations incorporating the aforementioned variables would allow for a more comprehensive evaluation of the determinants of vitamin D status and OSA severity in pediatric populations.

## 5. Conclusions

In conclusion, our study demonstrates that lower vitamin D levels and higher MPV are associated with OSA and its severity in normal-weight children. Additionally, vitamin D status was also associated with differences in sleep architecture, providing insight into potential underlying pathophysiological mechanisms.

While causality cannot be inferred, these markers may have potential utility in characterizing disease severity and informing individualized management.

Incorporating MPV and vitamin D assessments into the routine evaluation of pediatric OSA could enhance risk stratification. Future longitudinal and interventional studies are needed to validate these biomarkers and clarify their prognostic and therapeutic significance.

## Figures and Tables

**Table 1:** Main features of the OSA group according to disorder severity.

			OSA Severity		
	Control Group	Mild	Moderate	Severe	Overall *p*-Value *
N	138	32	46	60	
Age (years)	9.38 ± 1.23	9.38 ± 1.23	9.11 ± 1.43	8.72 ± 1.02	0.03
Sex, male (%)	56.5	58.6	52.6	51.3	0.56
BMI-SDS	0.62 ± 0.25	0.64 ± 0.22	0.65 ± 0.14	0.69 ± 0.13	0.13
			Laboratory findings		
Glucose (mg/dL)	89.15 ± 2.85	89.95 ± 2.83	92.04 ± 4.88	96.99 ± 1.95	**0.002**
Uric acid (mg/dL)	2.98 ± 1.31	8.84 ± 1.17	3.82 ± 1.63	5.35 ± 0.95	**<0.0001**
Vitamin D (ng/mL)	20.88 ± 2.23	20.66 ± 2.12	19.30 ± 4.68	15.45 ± 5.92	**<0.0001**
HOMA-IR	2.34 ± 0.39	2.32 ± 0.36	3.10 ± 1.07	4.41 ± 0.35	**0.0001**
ALT (UI/L)	22.63 ± 2.89	22.47 ± 3.02	26.71 ± 5.96	34.18 ± 2.05	**<0.0001**
AST (UI/L)	21.70 ± 3.10	21.76 ± 3.53	26.85 ± 7.36	35.99 ± 1.87	**<0.0001**
MPV	10.67 ± 1.24	10.42 ± 1.01	11.27 ± 1.52	12.26 ± 1.50	**0.003**
CRP, mg/dL	0.31 ± 0.24	0.30 ± 0.26	0.38 ± 0.30	0.62 ± 0.35	**0.0001**
ESR, mm/hr	1.70 ± 0.42	1.82 ± 0.49	2.04 ± 0.82	2.52 ± 0.93	**0.004**

A *p*-value < 0.05 was considered statistically significant, and values in bold denote statistical significance. * The “overall *p*-value” refers to the *p*-value from one-way ANOVA (for normally distributed variables) or the Kruskal–Wallis test (for non-normally distributed variables), comparing the three OSA severity groups (mild, moderate, and severe). Abbreviations: ALT, alanine transaminase; AST, aspartate transaminase; BMI, body mass index; CRP, C-reactive protein; ESR, erythrocyte sedimentation rate; HOMA-IR, homeostasis model assessment of insulin resistance; MPV, mean platelet volume; SDS, standard deviation score.

**Table 2:** GLM for analysis of variance of vitamin D in the study population.

	Study Population		
Source	Coefficient	F-Ratio	*p*-Value
Model		17.56	**<0.0001**
Sex	−0.01	−0.21	0.83
Age	0.06	0.65	0.51
BMI-SDS	0.07	1.42	0.15
HOMA-IR	−0.10	−1.96	**0.04**
OSA	−0.41	−3.93	**<0.0001**

Model r^2^ 0.24; adjusted r^2^ 0.23. A *p*-value < 0.05 was considered statistically significant, and values in bold denote statistical significance. Abbreviations: BMI, body mass index; HOMA-IR, homeostasis model assessment of insulin resistance; OSA, obstructive sleep apnea; SDS, standard deviation score.

**Table 3:** GLM for analysis of variance of vitamin D in the OSA group.

	Study Population		
Source	Coefficient	F-Ratio	*p*-Value
Model		12.91	**<0.0001**
Sex	−0.02	−0.39	**0.69**
Age	0.12	2.17	**0.003**
BMI-SDS	0.08	1.52	0.12
HOMA-IR	−0.26	−4.33	<0.0001
OSA severity	−0.22	−3.67	**<0.0001**

Model r^2^ 0.19; adjusted r^2^ 0.17. A *p*-value < 0.05 was considered statistically significant, and values in bold denote statistical significance. Abbreviations: BMI, body mass index; HOMA-IR, homeostasis model assessment of insulin resistance; OSA, obstructive sleep apnea; SDS, standard deviation score.

## Data Availability

The datasets generated and/or analyzed during the current study are not publicly available due to patient privacy and confidentiality concerns, but they are available from the corresponding author on reasonable request.

## Abbreviations

The following abbreviations are used in this manuscript:

AHI	apnea–hypopnea index
aOR	adjusted odds ratio
CRP	C-reactive protein
ESR	erythrocyte sedimentation rate
HOMA-IR	homeostasis model assessment for insulin resistance
MPV	mean platelet volume
ODI	Oxygen Desaturation Index
OSA	obstructive sleep apnea
